# Potential of Mineral Fraction in Compost-Like-Output, Methods of Its Obtaining and the Possibility of Using It in the Context of Circular Economy

**DOI:** 10.3390/ma13133023

**Published:** 2020-07-06

**Authors:** Jacek Połomka, Andrzej Jędrczak

**Affiliations:** 1 Regional Municipal Waste Treatment Plant in Marszów, 68-200 Marszów, Poland; 2 Institute of Environmental Engineering, University of Zielona Góra, 65-417 Zielona Góra, Poland; A.Jedrczak@iis.uz.zgora.pl

**Keywords:** municipal waste, MBT systems, CLO, glass recovery, mineral fractions

## Abstract

Most of the systems for the mechanical and biological treatment of waste used in Poland send the 0–80 mm fraction separated from the municipal waste stream, after biostabilization, entirely to a landfill. Such action is not in line with the adopted EU strategy focused on waste management in the circular cycle. The purpose of this work was to assess the technical feasibility of recovering the mineral fractions contained in compost-like-output (CLO) on the proprietary technological line designed for glass recovery. The research was launched in January 2019, and lasted for a subsequent 12 months. In the article, the amounts of mineral fractions possible to be separated from CLO are presented, as well as their morphological composition and selected properties being determined. The processing of CLO on the line allowed to recover on average 69.4 ± 7.0% of the glass. This product was accepted by glass recycling plants. Mineral fractions constituting waste from the glass separation process were tested for their use in winter road maintenance. Tests were also carried out confirming the possibility of using selected mineral fractions (0–10 mm) from CLO to obtain a waste cement mix useful for constructing road foundations using a standard amount of cement.

## 1. Introduction

In order to achieve the objectives of Directive 1999/31/EC in the area of reducing the amount of biodegradable waste disposed of in landfills at the beginning of the current century, the technology of mechanical and biological waste treatment (MBT) was developed [1,2]. This technology, focused on the biological stabilization of the organic fraction of municipal solid waste (OFMSW) before storage or for the preparation of MSW for combustion with energy recovery, has played a key role in the system of economy of MSW in Poland and in many other EU countries to this day [2].

At the end of 2016, there were 192 MBT systems in Poland with the processing capacity of around 11 million tons of waste per year. About 570 MBT systems with the processing capacity of 55 million tons of MSW were operated in Europe. It was anticipated that another 120 facilities with the total capacity of around 10 million tons/a would be commissioned by 2025. It was stated that the market position of MBT technology will continue to be strong in the coming years, although the pace of construction of these systems will clearly decrease [3].

For several years, the process of changing the waste management model from linear to cyclical has been underway to achieve sustainable development and increase Europe’s global competitiveness. The goal of a circular economy is to maintain the value of resources in the economy for as long as possible. In 2020, 50% of the mass of municipal waste generated should be recycled. It seems that this will be difficult to achieve, which is confirmed by the European Commission in a report prepared for the European Parliament, pointing out that 14 countries of the Community, including Poland, may not achieve a 50% recycling rate of municipal waste [4]. The new Directive of the European Parliament and of the Council of 30 May 2018 introduces new, higher targets for the recycling of municipal waste, assuming achieving recycling of mixed municipal waste at 55% in 2025, and reaching 60% in 2030, then 65% in 2035 [5].

Implementing a circular economy eliminates MBT technology from the market. However, MBT systems are and will be necessary to ensure the reduction in biodegradable waste storage for many years. They constitute a bridge between the current state of municipal waste management in Europe and the need to meet current needs, and the necessary intensive development of recycling. In the longer term, along with the irrevocable increase in material and organic recycling, MBT installations will be adapted to the growing supply of these streams of selectively collected waste.

A total of 12.5 Mt of municipal waste was generated and over 7.6 MSW Mt (over 60%) was directed to MBT systems in Poland in 2018. In the mechanical part of the installation, an organic fraction is separated on a sieve with 80 mm mesh (rarely 100 mm), which is most often stabilized under aerobic conditions. The compost-like-output (CLO) produced is practically 100% disposed of in landfills. Using an 80 mm sieve, the organic fraction constitutes approximately 48.5% of the mass of screened municipal waste [6]. This means that in Poland, approximately 3.5 Mt of the organic fraction coming from mixed municipal waste went to biological stabilization, resulting in approximately 2.45 million tons of CLO, which was removed at landfills.

Most installations recover secondary raw materials such as plastics (foil and PET), paper and cardboard, glass and fuel from waste [7], which are still present in significant quantities in MSW due to the low effectiveness of the selective collection system [8,9].

Only in a few systems, mineral fractions are recovered for material recycling. For example, in one MBT plant, stones, glass and ceramics are separated. This mineral fraction is further processed mechanically in another external installation and then recycled, e.g., for road construction [10]. Research is being conducted regarding the finding of possibilities of using the final products of MBT systems, including separated material fractions from CLO [11,12,13].

**Figure 1:**
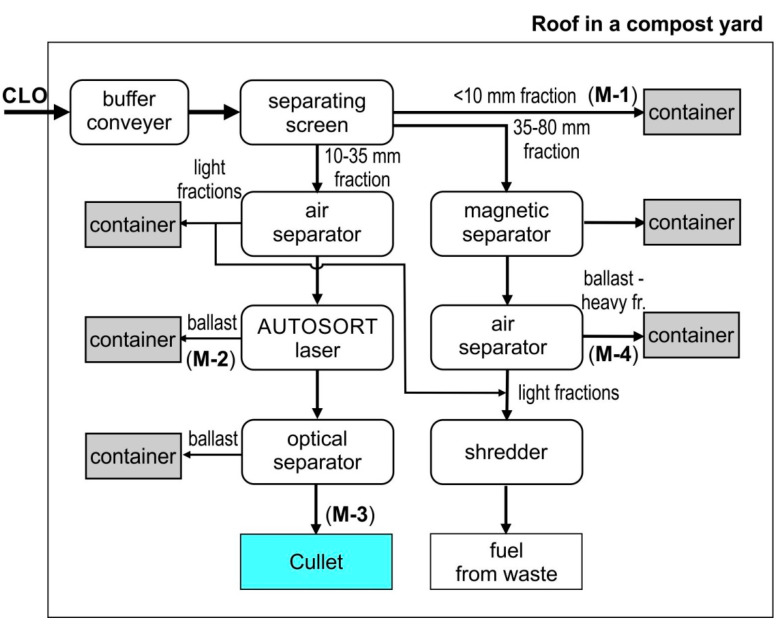
Technological scheme of the compost-like-output (CLO) processing line.

At the end of 2018 at the waste management plant in Marszów in the province of Lubuskie, the first stabilization processing system in Poland was commissioned (Figure 1). Its development was associated with attempts to extract packaging glass from CLO and achieve such parameters of recovered glass that would allow it to be recycled.

The purpose of this work was to assess the technical feasibility of recovering mineral fractions contained in CLO on a process line designed for the purpose of glass recovery, using processes commonly used in MBT systems. Types and amounts of mineral fractions possible to be separated from the CLO are presented, as well as their morphological composition and selected properties and the results of research on their use were determined.

## 2. Materials and Methods

The research on the content of the mineral fraction in CLO focused on four types of waste generated on this line: 0–10 mm fraction (waste M-1), 10–35 mm fraction remaining after separation by Tomra Sorting Autosort Laser (waste M-2), 10–35 mm fraction after Combisense photo separator (waste M-3) and a heavy fraction, 35–80 mm, after the NIHOT air separator (waste M-4).

The tests were carried out in the period from 15 January 2019 to 21 January 2020. A total of 29 measurement series were performed. In 23 series, the CLO manufactured in the MBT system in Marszów was processed, and in 6 series, the CLO was supplied from other MBTs located in various regions of Poland (Gdańsk, Kryniczna, Ścinawka, Dąbrowa Górnicza, Stargard and Piotrowo Pierwsze).

The MBT plant in Marszów processes waste from 22 communes, inhabited by over 200,000 people. In the mechanical part of the MSW system, after removing obstructing waste, after passing through the sack burner, the waste stream is separated in an 80/280 mm drum screen into 3 fractions. Fractions > 280 mm and 80–280 mm are subjected to treatment directed at the production of fuel from waste. Sub-fraction <80 mm is given biological stabilization under aerobic conditions. The process is carried out in two stages. Stage I is an intensive stabilization of waste in reinforced concrete bioreactors, with fully automatic control of its course, with a duration of 3 weeks. Stage II is the maturation of waste in piles in the open ground for a period of 10–12 weeks, with waste transfer [14].

The CLO processing line is comprised of 5 basic machines.

**Flip-Flop Screening Machine** (IFE Aufbereitungstechnik GmbH, Waidhofen an der Ybbs, Austria), with a flexible screening deck, on which the stabilized compost is separated into three fractions: 0–10 mm (M-1), 10–35 mm and 35–80 mm. The technical data of the unit are available on the manufacturer’s website www.ife-bulk.com/de/.

**Figure 2:**
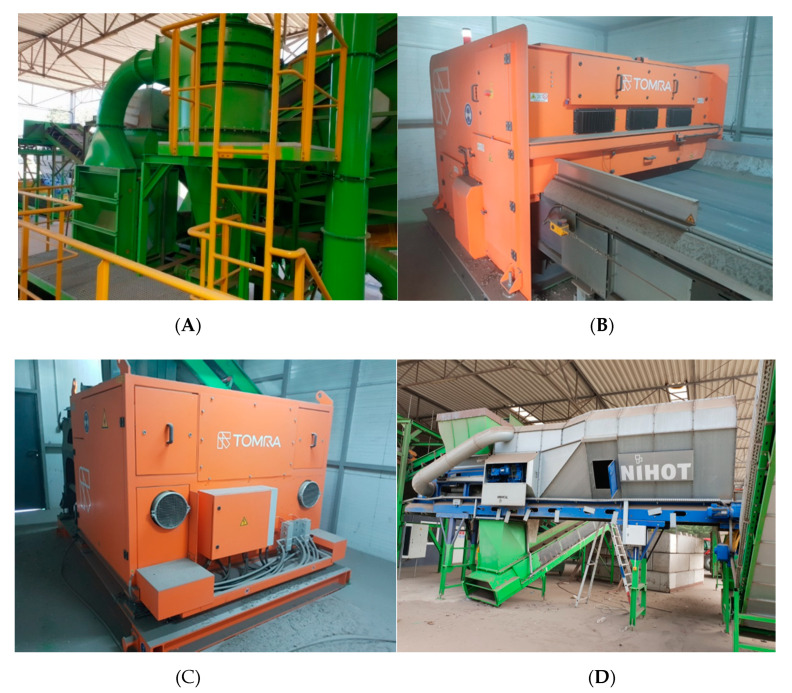
Machines used for the separation process: (**A**) ZIG ZAG Air Separator, (**B**) Autosort Laser Separator, (**C**) Combisense Chute Optical Separator, (**D**) NIHOT Air Separator.

**ZIG ZAG Air Separator** (Trennso-Technik Trenn- und Sortiertechnik GmbH, Weißenhorn, Germany), (Figure 2A), in which the 10–35 fraction is divided into a light fraction and a heavy fraction. Screened material is introduced into the charging channel in a zigzag shape and distributed over its entire surface. The air pumped by the fan flows through the separator from the bottom upwards and blows out the light parts from the feed material. The technical data of the unit are available on the manufacturer’s website: www.trennso-technik.de.

**TOMRA Autosort Laser** (Tomra Sorting GmbH, Mülheim-Kärlich, Germany). The Autosort Laser unit, a combination of NIR technology and advanced laser technology, enables the detection of glass and its separation from other waste such as stones, ceramics, debris, metals (M-2) and polymers, including transparent ones. When the sensors detect the material to be sorted, they send a signal to the control unit to release the corresponding valves in the valve strip at the end of the conveyor. The detected material is separated from the rest by compressed air streams. The technical data of the unit are available on the manufacturer’s website: www.tomra.com (Figure 2B).

**The TOMRA COMBISENSE CHUTE Optical Separator** (Tomra Sorting GmbH, Mülheim-Kärlich, Germany) is equipped with a high-resolution scanning camera with color detection and an electromagnetic (EM) sensor. The batch material is evenly fed into the vibrating chute, where it is analyzed by the scanning camera. If the sensor detects the material to be sorted, it sends a signal to the control unit to release the corresponding valves in the valve strip at the end of the chute. The detected material is separated from the rest by compressed air streams. The sorted material is divided into two fractions in the separation chamber: glass concentrate (M-3) and ballast. The technical data of the unit are available at www.tomra.com (Figure 2C).

**NIHOT Air Separator** (Nihot Recycling Technology B.V., Amsterdam, The Netherlands), in which the input material is separated into the heavy fraction (M-4) and the light fraction through a separating drum and air stream. Technical data of the device are available on the manufacturer’s website www.nihot.co.uk (Figure 2D).

A detailed description of the CLO processing line and the equipment used is presented in the publication [13]. Waste after intensive stabilization was sent for tests. In the last week of stabilization, the parameters of this process were set at such a level that intensive waste drying took place.

Waste supplied from other MBT plants also constituted the <80 mm fraction after intensive oxygen stabilization. For these plants, the areas of collection of MSW are different, as are the devices used and the parameters of the biological stabilization process, and as a result of which, the characteristics of the samples tested were also different.

The waste streams separated on the line were analyzed by determining their morphological composition as well as humidity and roasting losses. The scope of morphological analysis included division of waste into organic (food waste, green and garden waste, wood and other organic waste types), paper, plastics, glass, metals, inert (debris, stones) and others. The analyses were carried out in the factory laboratory of ZZO Marszów and periodically, for control purposes, in the Laboratory of the Institute of Environmental Engineering at University of Zielona Góra, which is accredited for taking and analyzing waste samples. Waste analyses were performed according to the standards and procedures in force in the accredited laboratory [15,16]. Analyses within the scope of studies concerning the use and application of mineral fractions obtained from stabilized compost in the construction industry were performed in an accredited construction laboratory in Zielona Góra.

The characteristics of the waste samples tested are presented in Table 1.

The weight of processed samples ranged from 28.4 to 154.5 tons and averaged 47.8 ± 26.8 tons. Samples from ZZO Marszów were very well dried. Their humidity ranged from 6.0 to 15.5% (average 10.0 ± 2.6%). CLOs from ZZO Marszów contained 51.4 ± 6.7% fraction <10 mm, 9.1 ± 1.7% debris and stones and 17.4 ± 2.1% glass.

The weight of delivered samples ranged from 10.3 to 22.0 tons and averaged 17 ± 5.0 tons. The humidity of entrusted samples was more diverse and ranged from 9.6 to 32.0% (average 20.9 ± 9.6%). These CLO samples contained 52.5 ± 13.3% of fraction <10 mm and 11.9 ± 5.8% of debris and stones. The average share of glass in waste from other plants was 1.5 times lower (11.4 ± 2.4% of glass).

## 3. Results

### 3.1. Mineral Products Derived from CLO

The work of the CLO treatment line was evaluated in terms of content and level of material recovery (*η*) in the product (glass fraction). Material recovery is the quotient of its quantity in the product in relation to its content in the feed (CLO). The results are shown and discussed for the intermediate product after each device to show the effect of each step of the process carried out.

**Figure 3:**
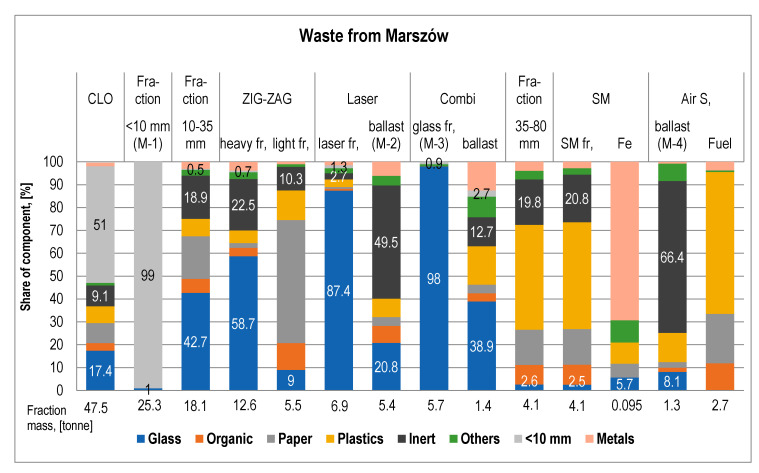

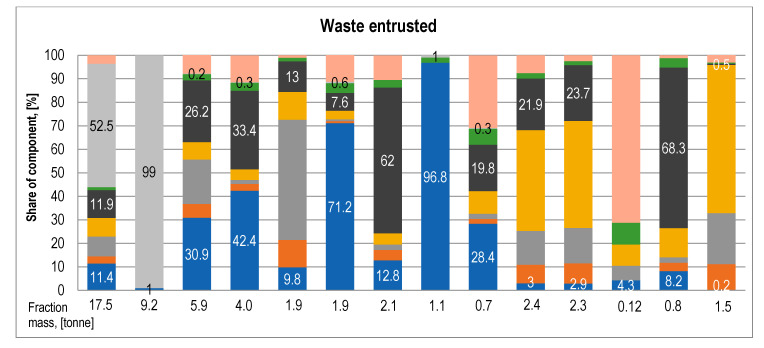
Mass and material composition of intermediate products obtained in subsequent stages of the stabilization process from Marszów and entrusted from other mechanical and biological waste treatment (MBT) systems on CLO.

**Figure 4:**
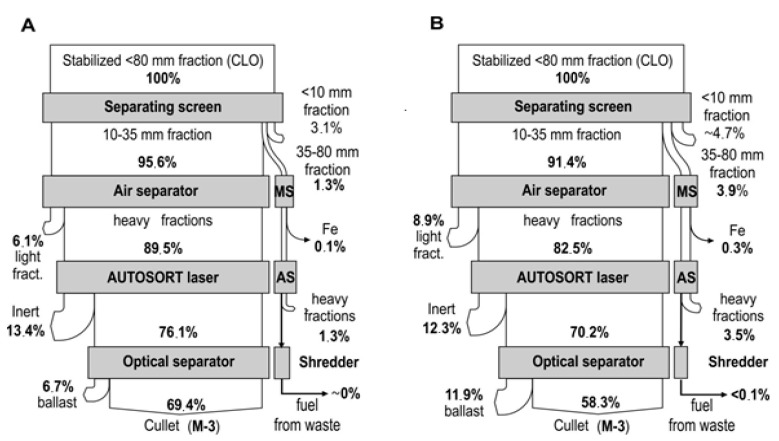
Average levels of debris and stone recovery (inert) from CLOs in subsequent stages of their treatment process ((**A**)—waste from Marszów, (**B**)—entrusted waste).

The mass of intermediate products obtained during the processing of CLOs from Marszów and entrusted from other MBT systems on the CLO processing line and their material composition are presented in Figure 3. Figure 4 presents the average levels of debris and stone recovery (inert) from CLOs in the subsequent stages of the glass concentrate production process (A—waste from Marszów, B—entrusted waste) and Figure 6 presents that of glass.

The VARIOMAT dual-level screen separates the CLO stream into three fractions: <10, 10–35 and >35 mm.

Fraction <10 mm constituted 50.9 ± 6.4 and 53.0 ± 13.5% of the mass of CLOs, respectively. It was a mixture of ash and sand, as well as, in large quantities, an organic fraction that was dried and largely ground to grain <10 mm during the unloading of chambers, transport of CLOs to the screen and screening operations. The high content of roasting losses confirms the high content of organic matter in this fraction (from 27.1 to 45.8% sm, 36.8% sm on average).

The 10–35 mm fraction directed to the production of glass concentrate constituted 39.7 ± 5.3% of the mass of CLOs from Marszów and 34.5 ± 6.7% of the mass of entrusted CLOs.

The fraction share > 35 mm in the mass of CLOs was small—on average 9.4 ± 2.2% of the sample from Marszów and 12.5 ± 9.9% of the sample entrusted. Mainly plastics passed into this fraction, on average at 57.6 and 63.7%, metals at 28.0 and 23.4%, other impurities at 21.0 and 21.6% and inerts (stones, debris) at 20.7 and 21.4%, respectively, and in the case of waste entrusted organic fraction loss, 30.6%.

The first mineral fraction is <10 mm (M-1). The mineral nature of this fraction was confirmed by industrial tests to separate this waste into organic and mineral fractions and to determine the actual amount of minerals for potential use. The first involved the use of air separation on Trenso separation tables.

Fraction <10 mm was fed to a vibrating screen driven by an eccentric. The inclination of the sieve can be adjusted steplessly. The air is introduced into the fractionated product by means of a pressure fan under the screening deck. This fluidizes the light elements and separates them from the heavy elements. The heavier parts are moved up the table, and the lighter parts are transported down towards the outlet of the light fractions, depending on the movement and the angle of inclination of the screening deck. Results obtained in the studies: 40.7% mineral fraction (including fraction <5 mm —32.9%, >5 mm stones—5.2% and glass—2.6%), light fraction 50.6% and dust 8.7%. Tests were also performed on Doppstadt water separators. The tests were carried out at BYŚ in Warsaw in July 2019. The share of the heavy mineral fraction in the <10 mm fraction was 51.2%.

Two more mineral fractions constitute ballast (M-2) and ballast (M-4), which in material terms are a mixture of stones and debris contaminated with various components. M-2 waste is a fraction separated on the LASER separator from the 10–35 mm fraction after removing paper, organics and plastics on the air separator. M-4 waste is a heavy fraction separated on an NIHOT air separator from the 30–80 mm fraction after removing metals from it on a magnetic separator.

Material balances of debris and stones (inert) in the treatment processes of the CLO produced in the system in Marszów and entrusted CLOs are presented in Figure 4.

**Figure 5:**
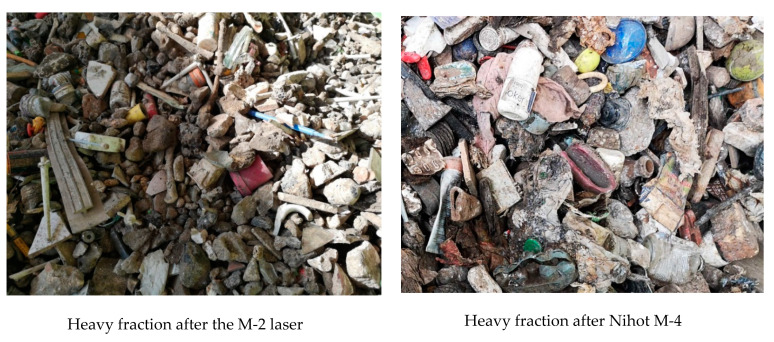
Rubble and stone fractions (inert) from CLOs after the M-2 laser and after the Nihot M-4 separator.

In the case of ZZO in Marszów, the M-2 fraction after the Laser autosorter (Figure 5) was 11.5 ± 2.8% of the CLO weight. This waste constitutes debris and stones (49.5 ± 10.7%) and glass (20.8 ± 6.4%). Up to 62.7% of the stones and debris contained in the CLO passed through (Figure 4). Contamination consists primarily of paper (8.1 ± 2.7%) and organic contamination (7.5 ± 2.5%) (Figure 3). The waste showed a low humidity of 4.9 ± 1.2% and low roasting loss of 14.8 ± 9.7% (laboratory analysis). The share of minerals determined in industrial tests on water separators by Doppstadt was 70%. In the case of entrusted CLOs, the M-2 ballast constituted 11.3 ± 6.5% of the CLO mass. A total of 4% less stones and debris contained in the CLO passed into it than in the case of waste from Marszów. It contained more debris and stones (62.0 ± 6.6%), but significantly less glass (12.8 ± 5.3%). The paper content was 4.8 ± 3.6% and organics was 4.4 ± 3.3% (Figure 3).

The M-4 ballast (Figure 5) separated from the 35–80 mm fraction constituted 3.2 ± 0.9% of the mass of processed CLO from ZZO in Marszów. Up to 20.5% of the stones and debris contained in the CLO passed through (Figure 3) and their share in waste was 62.2 ± 6.6%. The waste contained 12.8 ± 5.3% glass and 10.4 ± 1.2 metals. Plastics constituted 4.8 ± 3.6% of its mass and organics constituted 4.4 ± 3.3% (Figure 3). The waste showed a low humidity of 8.4 ± 5.9% and low roasting loss of 13.3 ± 8.1% (laboratory analysis). In the case of CLO M-4 ballasts after the autosorter, the laser constituted 4.2 ± 3.9% of the CLO mass. A total of 24.9% of all stones and debris passed into it. This waste contained 68.3 ± 1.8% of debris and stones and only 3.6 ± 3.8% of glass. Among organic contamination, the share of plastics was 12.4 ± 3.1% (Figure 3).

**Figure 6:**
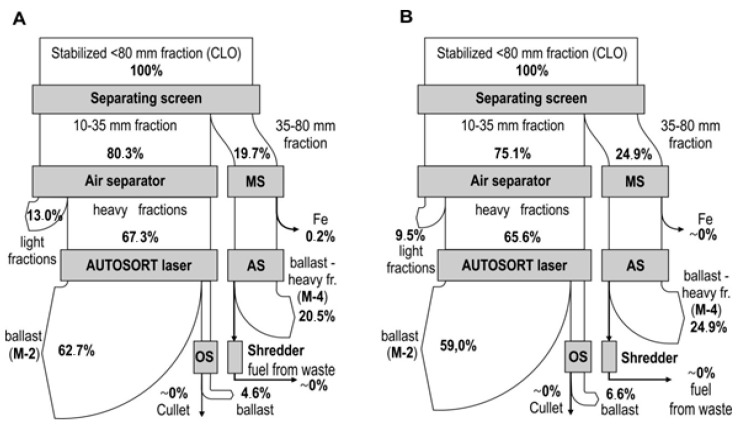
Average levels of glass recovery from CLOs in subsequent stages of their treatment process ((**A**)—waste from Marszów, (**B**)—entrusted waste).

The material balance of glass in the treatment processes of the CLO produced in the system in Marszów and entrusted CLOs are presented in Figure 6.

The glass fraction (M-3) was 11.7± 3.4% of the CLO mass in the case of waste from ZZO in Marszów, and 7.8 ± 2.8% in the case of entrusted CLOs.

**Figure 7:**
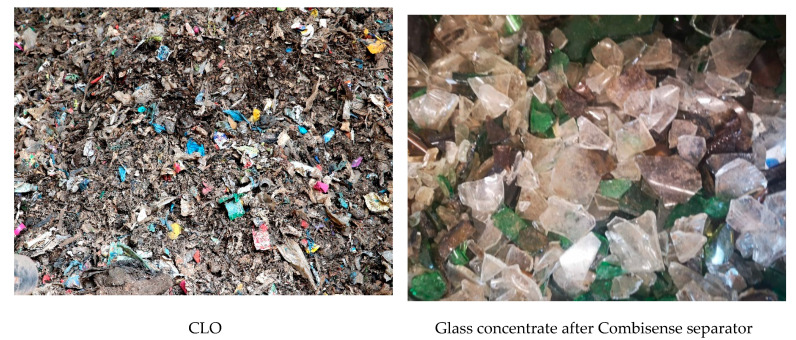
Fractions after subsequent stages of the glass recovery process from CLOs.

Figure 7 presents photos of CLO and glass fraction after subsequent stages of the glass recovery process.

The processing of CLO on the line allowed to recover from 49.8 to 74.3% of the glass it contained (on average 69.4 ± 7.0%) for samples from Marszów and from 38.3 to 72.1% (on average 58.3 ± 14.2%) for samples from other MBT systems. The concentrates contained practically no paper, plastics or metals. Organic contamination was only present in two samples from Marszów in trace amounts of 0.1 ± 0.1% (Figure 3). Fraction <5 mm was present in four samples from Marszów in an amount of 0.2 to 2.5% and in one sample from other MBT systems (5.8%). The main impurity in the glass concentrate found in all samples was “other ingredients”. Their share was on average 0.9 ± 1.0% in samples from Marszów and 2.2 ± 2.0% in entrusted samples. The content of glass in the final product ranged from 93.0 to 99.5 (on average 98.0 ± 1.9%) of the fraction weight in the case of CLOs from Marszów and from 92.8 to 99.1% (on average 96.8 ± 2.8%) in the case of CLOs from other MBT systems (Figure 3). In 14 out of 30 measuring series carried out, the share of glass was 99%. Glass contents lower than 98.0% were found only in six studies. These were samples obtained from high-hydration CLOs.

### 3.2. Application of the Mineral Fraction Obtained from CLO

The 0–5 mm fraction was tested for the possibility of using it for winter road maintenance. The grain composition was tested by a screening aggregate for concrete according to PN-EN 933-1 [17] and the following results were obtained: dust <0.063 mm—12.5%, main fraction/0–2.0 mm/ = 89.6%, oversize = 10.4%. Based on the current requirements for tenders based on European standards for sand for winter maintenance, the screening is correct. Unfortunately, this aggregate was too light, it was impossible to test the pen indicator and it contained a lot of organic parts. Comparison of the costs of purchasing rinsed sand meeting the requirements and preparing the material from the CLO was more favorable for sand. Therefore, further research in this direction was abandoned, also taking into account the problem in acquiring potential customers who would like to use the material obtained from waste for winter road maintenance.

**Figure 8:**
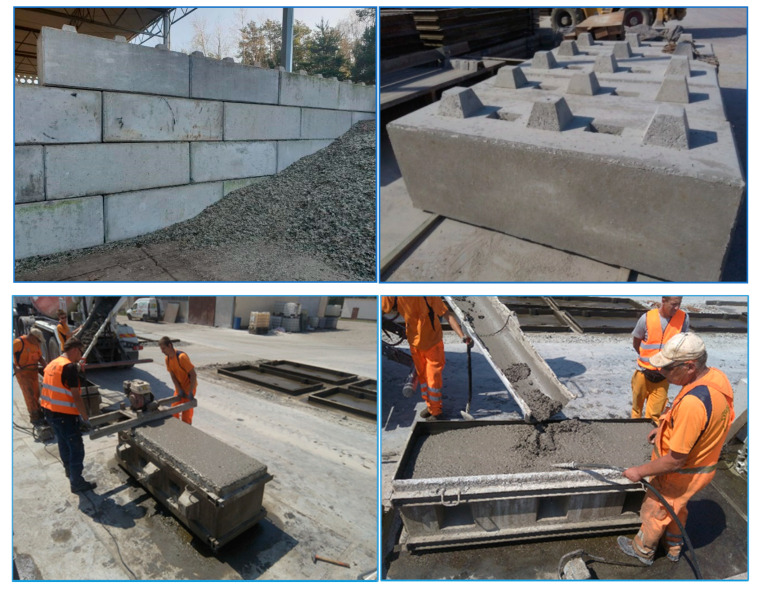
Currently, the blocks are used successfully in ZZO Marszów.

A C25/30 concrete mix formula was prepared, Portland cement CEM I 42,5 according to PN-EN 206:2014 [18], in which the primary aggregate with granularity of 5–10 mm was replaced by mineral waste of the same fraction, but originating from CLO. At the beginning, the prepared formula was tested in a laboratory, testing the compressive strength of concrete according to PN-EN 12390-3:2011 [19] along with PN-EN 12390-3:2011/AC:2012 [19]. Samples after 56 days showed an average compressive strength of 40.5 MPa. The obtained result was used to assess the identity of the concrete class according to PN-EN 206+A1:2016-12 [18], which showed that the developed formula for a mixture of concrete with an aggregate derived from CLO meets the standards of class C25/30. Concrete absorbability tests according to PN-88/B-06250 and tests of water permeability through concrete according to PN-88/B-06250 [20] were also carried out, which confirmed that the concrete mix meets the standards for class C25/30. The formula prepared in this way was handed over to a local concrete company and a trial batch was commissioned in the amount of 100 concrete blocks for retaining walls and boxes. The blocks made were used to make storage boxes at ZZO Marszów (Figure 8). As part of these activities, the possibility of using this mineral fraction from CLO on an industrial scale was confirmed, providing the possibility of rational use of natural resources in the form of an aggregate.

As part of the laboratory tests of the mineral aggregate obtained from CLO with granulation of 0/31.5 mm, the following procedures were performed: determination of the grain composition according to PN-EN 933-1 [17], he sand index according to PN-EN 933-8+A1:2015-07, determination of density and water content by the Proctor method according to PN-EN 13286-2:2010 [21], determination of resistance to comminution by the Los Angeles method according to PN-EN 1097-2:2010 [22] and determination of aggregate frost resistance according to PN-EN 1367-1:2007 [23], in order to assess the suitability of the material for incorporation as a hardening layer on communal dirt roads and access roads after mechanical stabilization or as material bound with hydraulic binders. The results are shown in Table 2. Due to the lack of general requirements for these type of roads, the scope of the tests was established on the basis of requirements for mixtures not bound to the improved subsoil, substructures and pavements presented in the following documents: WT-4 technical requirements “Unbound mixtures for national roads” and WT-5 technical requirements “Hydraulic binder mixtures for national roads” of the General Directorate for National Roads and Motorways (GDDKiA).

The screening practically met the requirements of the WT-4 GDDKiA document containing the requirements for aggregates for unbound aggregate pavement, as the sand index test was positive with a result above 35%. This material has low resistance to grinding and low resistance to freezing and thawing in the presence of water. With the right approach of investors to the expected parameters of the material, it can successfully fulfil its role in the intended applications.

Test mixes were also made with hydraulic binders: Portland cement CEM I 42.5R and metallurgical CEM III 32.5 N and a mixture of fly ash from the Konin power plant with the fraction of 0/1 mm. Formulas have been prepared without increasing the binder content compared with standard formulas with aggregates of natural origin. Compressive strength tests of bound mixtures based on various types of cements and cement–ash mixtures provided results as expected, allowing for obtaining the expected classes of compressive strength after 28 days of maintenance (in the range of 2.5–7.0 MPa). Tests of freezing and thawing strength of samples confirmed low aggregate frost resistance, giving unsatisfactory results, i.e., frost resistance index below 0.6. The foundation layer made of cement bound aggregate after several freezing–thawing cycles in the structure will degrade and begin to behave as an unbound aggregate.

At the beginning of 2019, research was also carried out to obtain an answer as to the possibility of using selected mineral fractions (0–10 mm) from CLO to obtain a waste cement mix useful for constructing road foundations using a standard amount of cement and compaction energy. The quality criteria set by the PN-S-96012:1997 [24] standard were adopted as the assessment criteria when making and receiving the foundation made of cement-stabilized soils and the subsoil layer improved with cement for the road surface. The composition of the mix as well as the preparation and sample testing were carried out in accordance with the procedures contained in the PN-S-96012:1997 [24] standard. The optimum composition of the mixture was determined through mixing waste with cement in the amount of 6 to 10% of the weight of waste and water, in an amount such that the waste cement mix has the ability to form, i.e., the ability to preserve the shape after compaction using a standard amount of compaction energy and maximum density after compaction. A 10% cement mix based on the weight of the waste and 10% water in the mix (based on the weight of the waste and cement) was considered optimum and a full range of compression strength tests were carried out using it. The density of the cement–waste mixture was defined with a determined humidity of 10% at the level of 1958 kg/m^3^. The frost resistance index, defined according to PN-S-96012:1997 [24], could not be determined as the ratio of the strength of 28-day-saturated samples subjected to 14 freezing and thawing cycles to the strength of 28-day-saturated samples, expressed as a decimal fraction. The reason was that such a large frost destruction of samples after four cycles makes it impossible to carry out strength tests. The properties required by the PN-S-96012:1997 [24] standard and many years of road construction practice for layers used in road pavement construction, which could potentially be carried out using a waste cement mix, are presented in Table 3.

The data in Table 3 show that the waste-based mixture does not meet the required parameters. Better parameters of the waste cement mix can be achieved by increasing the mix’s ability to compact and wedge by modifying the shape and size of the waste grains, optimizing the grain composition of the waste mix and increasing the amount of cement. However, the required significant percentage of cement in mixtures, close to the maximum amount in terms of price and technological reasonability, disqualifies cement–waste mixtures from being used commercially.

**Figure 9:**
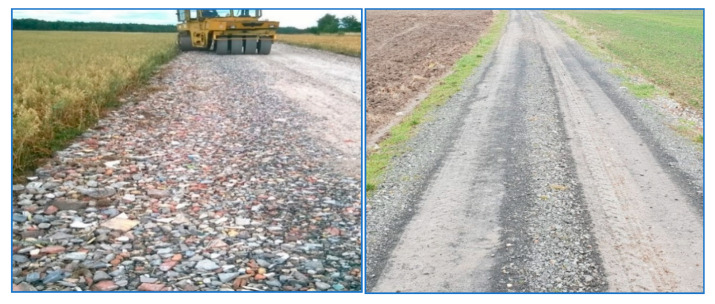
Road surface during repair and currently.

In September 2018, a 900 m long municipal road connecting the village of Marszów with the system was rebuilt using the mineral fraction obtained from the CLO (Figure 9). The reconstruction consisted of road trenching, fertilizing the mineral fraction from the 0–80 mm CLO with a thickness of 20 cm after compaction and then applying a top layer of 0–31.5 mm granite aggregate with a thickness of 10 cm after compaction.

## 4. Conclusions

The processing of the CLO on the designed line allowed to recover on average 69.4 ± 7.0% of the glass (M-3) it contained in the case of samples from Marszów and on average 58.3 ± 14.2% in the case of samples from other MBT installations. In 14 out of 30 measuring series carried out, the share of glass was ≥99%. Glass contents lower than 98.0% were found only in six studies. These were samples obtained from high-hydration CLOs. This product was accepted by glass recycling plants due to the low degree of contamination with other materials and the appropriate particle size.

The obtained test results allow to state that the aggregate obtained from the CLO at ZZO (Zakład Zagospodarowania Odpadów Sp. z o.o.) in Marszów (fractions M-1, M-2 and M-4) may be a suitable material for the intended use in the mechanical hardening of local dirt roads, assuming an appropriate executive regime and constant supervision over the maintenance of completed roads. In addition, it is possible to use fractionated aggregates from the CLO to replace natural aggregates in concrete products. However, from an economic point of view, at current aggregate prices and the cost of obtaining a 5–10 mm fraction from CLO, its use in concrete elements is not justified. Therefore, the use of the mineral fraction derived from the CLO to harden local dirt roads is reasonable in terms of costs and reductions in shrinking natural resources, and also perfectly fits into EU directives on waste recycling.

## Figures and Tables

**Table 1:** Characteristics of processed waste, their humidity and morphological composition.

**No**	**MBT Plant**	**No. of Samples**	**Sample Mass**	**Humidity**	**Morphological Composition**							
					**Glass**	**Organic**	**Paper**	**Plastics**	**Inert**	**Other**	**<10 mm**	**Metals**
			**[Tonne]**	**[%]**	**The Share of Wet Weight, %**							
**I**	**Average values-samples of Marszów**	**23**	**47.8**	**10.0**	**17.4**	**3.3**	**8.8**	**7.3**	**9.1**	**1.2**	**51.4**	**1.5**
	Standard deviation		26.8	2.6	2.1	0.7	2.2	1.4	1.7	0.4	6.7	0.4
	Minimum value		28.4	6.0	14.0	1.6	3.7	3.6	5.4	0.6	39.5	0.9
	Maximum value		154.5	15.5	21.6	4.8	12.7	9.5	12.9	2.1	62.9	2.3
**II**	**Average values-samples entrusted**	**6**	**17.5**	**20.9**	**11.4**	**3.1**	**8.4**	**7.9**	**11.9**	**1.2**	**52.5**	**3.6**
	Standard deviation		5.0	9.6	2.4	1.4	2.4	4.7	5.8	0.5	13.3	1.3
	Minimum value		10.3	9.6	7.8	1.8	5.8	4.7	7.2	0.6	29.2	2.1
	Maximum value		22.0	32.0	14.6	5.5	11.2	16.9	23.4	1.9	67.4	4.9

**Table 2:** Results of fractional aggregates tests 0/31.5 mm.

No.	Indicator/Parameter	Standart	Result
1	Sand equivalent value	PN-EN 933-8+A1:2015-07	51.4%
2	Compared resistance to fragmentation LA	PN-EN 1097-2:2010	LA_70_
3	Compared resistance to freezing and thawing F	PN-EN 1367-1:2007	F_20_
4	Particle size distribution	PN-EN 933-1:2012	content of dust = 1.6% main fraction (0/31.5) = 79.9% oversize (≥31.5) = 20.1%
5	Density and water content—Proctor compaction	PN-EN 13286-2:2010	optimal humidity = 6.7% Max. dry density of solid particles *ρ* = 1.938 ton/m^3^

**Table 3:** Parameters of the cement waste mixture.

**No.**	**Mix Class**	**Compression Strength of Water Saturated Samples**		**Frost Resistance Indicator**
		**Rn7**	**Rn28**	
		**[MPa]**		
1	5.0	from 1.6 to 2.2	from 2.5 to 5.0	0.7
2	2.5	from 1.0 to 2.6	from 1.5 to 2.5	0.6
3	1.5	-	from 1.5 to 2.2	0.6
Property of cement–waste mix		0.4	1.16	Total lack of frost resistance
